# Feasibility of a Therapist-Supported, Mobile Phone–Delivered Online Intervention for Depression: Longitudinal Observational Study

**DOI:** 10.2196/11509

**Published:** 2019-01-22

**Authors:** Philippe R Goldin, Riku Lindholm, Kristian Ranta, Outi Hilgert, Tiia Helteenvuori, Anu Raevuori

**Affiliations:** 1 Betty Irene Moore School of Nursing University of California Davis Sacramento, CA United States; 2 Meru Health Inc Palo Alto, CA United States

**Keywords:** cognitive therapy, depression, digital health, digital therapeutics, mindfulness, online intervention

## Abstract

**Background:**

Depression is a very common condition that impairs functioning and is often untreated. More than 60% of the treatments for depressive disorder are administered in primary care settings by care providers who lack the time and expertise to treat depression. To address this issue, we developed Ascend, a therapist-supported, mobile phone–delivered 8-week intervention administered at the Meru Health Online Clinic in Finland.

**Objective:**

We conducted two pilot studies to examine the feasibility of the Ascend intervention, specifically, dropout rates, daily practice, weekly group chat use, and changes in depression symptoms. We also explored whether daily practice and weekly group chat use were associated with changes in depression symptoms.

**Methods:**

A total of 117 Finnish adults with elevated depressive symptoms enrolled in Ascend, a program that included daily cognitive behavioral and mindfulness meditation exercises delivered through a mobile phone app, anonymous group chat with other users, and chat/phone access to a licensed therapist. Eight weekly themes were delivered in a fixed, sequential format. Depression symptoms were measured at baseline, every second week during the intervention, immediately after the intervention, and 4 weeks after completion of the intervention. Data were analyzed using intent-to-treat repeated-measures analysis of variance and linear regression models.

**Results:**

For studies 1 and 2, we observed dropout rates of 27% and 15%, respectively, decreasing daily practice and group chat use, and decreased depression symptoms from baseline to immediately and 4 weeks after the intervention (*P*<.001). We found that both more daily practice and chat group use predicted the occurrence of fewer depressive symptoms at 4 weeks postintervention (Study 1: *∆R*^*2*^=.38, *P*=.004 and *∆R*^*2*^=.38, *P*=.002, respectively; Study 2: *∆R*^*2*^=.16, *P*<.001 and *∆R*^*2*^=.08, *P*=.002, respectively).

**Conclusions:**

This therapist-supported, mobile phone–delivered treatment for depression is feasible and associated with reduced depression symptoms. Design features that enhance daily practice and group chat use are areas of future investigation. Validation of these results using a controlled study design is needed to establish the evidence base for the Ascend intervention.

## Introduction

### Background

Depression is one of the most common mental disorders, impacting more than 300 million people worldwide according to the World Health Organization (WHO) [1]. In addition, depression carries the heaviest burden of disability among all mental and behavioral disorders [2]. People who suffer from depression often experience significant problems with employment, physical health, interpersonal functioning, suicidal ideation, and suicidal attempts. Thus, depression inflicts a significant burden on the individual, the network of family and friends, and society at large.

There are several effective psychological [3,4] and pharmacological interventions [5] for depression. However, the WHO estimates that less than 50% of people with major depression receive any care for their symptoms. Many people with depression do not have access to effective clinical care and are not willing to use antidepressant medications. Others are hesitant to obtain mental health treatment due to individual and social stigma [6]. Furthermore, when treated with antidepressants, roughly one-third of patients do not experience significant alleviation in symptoms [7]. In primary care settings, only 20% of patients who are referred to psychotherapy follow up, and of those, more than 50% drop out of treatment [8]. Thus, from a public health perspective, there is a need to examine other ways of delivering interventions for depression that bypass these obstacles.

Online interventions for mental disorders, especially depression and anxiety, have gained increasing popularity during the last decade. These online interventions can be delivered in many different formats including psychoeducation, self-help programs, chat support groups, interactive health coaching, and professional-led online therapy [9]. Online interventions have the advantage of being accessible to patients who previously did not have access to such mental health care. Further advantages include their ability to be self-paced, be programmable to record practice, and have a flexible sequence of clinical content and exercise that can be tailored to the individual patient. Importantly, they are also able to support clinical techniques such as cognitive reappraisal and mindfulness meditation practices in real-time and natural contexts. Furthermore, there is evidence that web-based interventions that include human support or coaching increase adherence and produce better outcomes [10].

There is a growing body of evidence for the beneficial effect of online interventions for depression. A recent meta-analysis [11] of controlled trials from a 10-year period (2006-2016) showed that therapist-supported online cognitive behavioral therapy (CBT) and in-person CBT were equally effective in reducing depressive symptoms and superior to treatment as usual, waitlist controls, and attention controls. Furthermore, this meta-analysis did not find an interaction between depression severity and CBT efficacy, suggesting that therapist-supported online CBT may be helpful for patients at all levels of depression severity. A meta-analysis [12] of 15 randomized controlled trials (RCTs) demonstrated a significant impact of online mindfulness-based interventions on depression symptoms. Studies also suggest the cost effectiveness and cost utility [13,14] of online interventions for depression over traditional treatment modalities. Improved cost effectiveness combined with high scalability makes online interventions a promising area for development and dissemination of empirically supported interventions, especially for traditionally underserved patients.

More recently, the increased use and flexibility of mobile phones has presented a more promising route to extend the reach of clinical interventions. A recent meta-analysis involving 3414 participants across 18 RCTs of mobile phone–based interventions for depression [15] showed a small reduction in depression symptoms compared to an active control group (Hedge *g*=.22) and a moderate reduction compared to an inactive control group (*g*=.56). This study also found no statistical difference between the effect of mobile phone only and mobile phone plus human/computerized components for the treatment of depression. Furthermore, apps focused on mental health produced a greater reduction in depression symptoms than apps focused on cognitive training. Interestingly, the integration of specific features, including mood monitoring, CBT, and mindfulness, did not alter the observed effects sizes. In addition, a more conservative subanalysis of studies (n=16) that used intention-to-treat analyses or reported complete outcome data showed a moderate effect of these treatment methods on depression symptoms (*g*=.40). This meta-analysis suggests that mobile phone apps may be a potentially effective tool to introduce self-management skill training for adults with depression. However, we need to better understand the potential mechanisms (ie, mediators) and pretreatment patient features (ie, moderators) that predict outcome and help us refine mobile phone–delivered interventions for depression.

To address the public health need for more accessible interventions for depression, we developed a new mobile phone–delivered intervention that includes self-help modules, support from a personal remote therapist, and chat group access to an anonymous patient peer-group support.

### Present Study

The goal of this pilot study was to examine the feasibility of a newly developed mobile phone–delivered, therapist-supported 8-week intervention for depression in self-referred depressed individuals. We examined dropout, number of days of practice, and weekly use of an online group chat. For symptom reduction, we examined intervention-related changes in depression symptoms and whether daily practice and weekly group chat use predicted a reduction in depression symptoms. We proposed three hypotheses. First, we expected a dropout of ≤30% among all participants, daily practice on at least half of the days (ie, 28/56 days), and weekly group chat use for at least half (4/8) of the weekly group chat sessions. We also examined the pattern of change in daily practice and weekly group chat over the 8-week intervention. Second, we expected the Ascend program to be associated with significant decreases in depression symptoms. In study 1, we used the Beck Depression Inventory-II (BDI-II) [16] to measure depression symptoms. In study 2, we shifted to the Patient Health Questionnaire 9-item version (PHQ-9) [17], because it is more commonly used than BDI-II in primary health care. Third, we expected that greater amount of daily practice and weekly group chat use during the 8-week intervention would predict reductions in depression symptoms.

## Methods

### Recruitment

For studies 1 and 2, participants were recruited from March to September 2017 in Finland via online Facebook advertisements for the Meru Health Online Clinic, a national remote health care provider approved by the Finnish National Supervisory Authority for Welfare and Health (Valvira approval number V/25535/2017). Meru Health operates under HIPAA (Health Insurance Portability and Accountability Act); legislation and all protected health information is stored in a HIPAA-compliant cloud storage hosted by a company called Datica [18]. All data are encrypted in transit, end to end, and at rest.

Participants were offered a free online intervention for depression that included mobile phone–delivered content, anonymous weekly chat group, and private phone/chat access to therapists. All participants were required to have a mobile phone. To enter the study, participants had to demonstrate some depression symptoms based on the BDI-II (>9) in study 1 and at least mild depression symptoms based on the PHQ-9 (>4) in study 2. We increased the depression symptom cut-off in study 2 to match the validated score of PHQ-9 for screening mild depression. Other inclusion criteria included the perceived ability of the participant by both the participant and therapist to commit to an 8-week online intervention with a minimum of 20 minutes of practice 6 days per week. The exclusion criteria included previous suicide attempts, severe suicidal ideation with a specific plan, severe self-harm, active substance abuse, and a history of psychotic disorder.

### Procedures

We informed participants that the goal of the study was to examine an online intervention for depression, delivered through a mobile phone app. Participants provided informed consent through the Ascend mobile phone app for their anonymous data to be used for further intervention refinement and research purposes. Participants were not compensated for their participation. They enrolled in the program at no cost. Institutional review board exemption was granted by Pearl IRB (Indianapolis, IN) for analyses of previously collected and deidentified data.

We provided participants a link to download the Meru Health Ascend app on their mobile phone to access the intervention content. Participants used a unique identification number to record depression symptoms at baseline, during the intervention, immediately after the intervention, and 4 weeks after the intervention. We trained participants on how to access an anonymous chat group and converse with the study therapist via direct one-to-one chat messaging or, in few exceptions, phone calls.

As part of the standard treatment procedure at the Meru Health Online Clinic, Ascend study therapists conducted phone interviews to examine inclusion and exclusion criteria before enrollment and determine participant suitability for the online intervention. After the program, the study therapist spoke to participants via phone calls to assess the participant’s experiences and address any further needs. In addition, participants had an opportunity to speak directly with the therapist via phone calls during the program. This occurred in only a few cases when a participant had a specific concern or question that could not be properly communicated by chat messaging with the study therapist.

### Ascend Program

The Ascend program consists of 8 modules delivered in a fixed order through a mobile phone app over an 8-week period, including practices derived from mindfulness-based stress reduction [19], mindfulness-based cognitive therapy [20], CBT [21], and behavioral activation therapy [22] treatment protocols. Modules are delivered each week in the following sequence: Introduction to mindfulness, Low mood and motivation, Self-compassion, Managing worry, Overcoming thinking traps, Rethinking your life values, Being aware of your relationships, and Relapse prevention. The content was designed to teach participants skills based on mindfulness meditation and cognitive behavioral therapy. The content for each week unlocked automatically during each of the 8 weeks of the Ascend intervention without the need to view prior content and complete prior exercises. The Ascend program has high standards of data security and adheres to the European Union General Data Protection Regulation requirements (EU 2016/679). If there were any signs of mental state deterioration during the intervention, the study therapist conducted an additional phone-based assessment of the participant’s condition. For emergencies such as severe suicidality, the Ascend intervention includes a written security plan that all participants reviewed with the therapist before starting the program.

The Meru Health therapists provided ongoing individual support as needed and curation of the group chat during the Ascend intervention. The interaction took place primarily via chat messaging and, in a few instances, phone calls. Therapists included one medical doctor and two master’s-level clinical psychologists, each of whom had training in mindfulness-based stress reduction or mindfulness-based cognitive therapy. The patients could reach out to their therapist whenever they wanted. However, more than 50% of the communication was initiated by the therapist, not the patient. The therapist would normally check in with the patient via chat at least 2-3 times/week to see how the patient was doing. On an average, the therapist spent 20 minutes on each patient per week, including time spent chatting with the patient and reviewing the patient’s data and progress.

Study therapists used a Professional Dashboard (Meru Health Inc, CA), which is a secure web-based tool to monitor participant progress and chat with participants. The mobile phone app hosted all the weekly program content, including text, video, audio, and graphics. The intervention included video lessons every week, audio-guided mindfulness-meditation practices, visual graphics that illustrated cognitive-behavioral principles, and journaling prompts.

The mobile phone app consisted of the following components: a “Me” screen to access the daily practices and learning materials; a “Program” screen to view the structure of the whole Ascend program, identify where on the timeline one is currently located, and access already completed modules and practices; a “Group” screen to view other anonymous participants’ written reflections on different practices and lessons (the Group screen did not allow any commenting, but rather only reading of therapist’s and other participants’ comments); a “Notifications” screen to track therapists’ messages and newly available lessons or practices; and an “Other” screen to view the Emergency Plan, Privacy Policy, Terms of Use, and a single button to contact the therapist via chat messaging.

### Measures

We used multiple measures to assess the feasibility of the Ascend intervention. We measured dropout from the intervention, operationally defined as less than 4 weeks of active participation during the 8-week intervention. We also measured completion of mobile phone–delivered practices (number of days during the 8 weeks) and participation in the once-weekly chat group (a binary yes/no measure). As stated above, we shifted from the BDI-II in study 1 to the PHQ-9 in study 2 to measure depression symptoms, because it became clear that the latter self-report measure is more commonly used across a wide variety of health care settings.

### Depression Symptoms

#### Beck Depression Inventory-II

Depressive symptoms were measured using the BDI-II, a scale containing 21 items rated 0-3 points in terms of intensity, with total scores ranging from 0 to 63. BDI-II scores of 14-19 points suggest mild depression; 20-28 points suggest moderate depression, and 29-63 points suggest severe depression. To enter the Ascend intervention, the participant had to score a minimum of 10 points at baseline. The BDI-II has demonstrated high internal consistency (Cronbach alpha=0.9) in outpatient samples [23].

#### Patient Health Questionnaire-9

The PHQ-9 is the 9-item depression scale extracted from the full PHQ. Because each of the 9 items can be scored from 0 (not at all) to 3 (nearly every day), the PHQ-9 score can range from 0 to 27 points. PHQ-9 scores of 5-9 points suggest mild depression, 10-14 points suggest moderate depression, 15-19 points suggest moderately severe depression, and 20-27 points suggest severe depression. To enter the Ascend program, the participant had to score a minimum of 5 points at baseline. Prior large-scale studies have shown that the PHQ-9 has excellent internal reliability, with Cronbach alpha of 0.89 in primary care settings, and excellent test-retest reliability [24].

#### Therapist Rating Questionnaire

To assess the patient-therapist interaction, we used a single-item question: “How valuable has the therapist interaction been for you?” This rating ranged from 1 to 5, with higher values indicating greater value. The patient-therapist interaction was assessed at weeks 1, 3, and 6 and after the intervention.

### Statistical Analysis

We computed descriptive statistics for different indicators of intervention engagement. We plotted the number of days per week of practice during the intervention and the number of weeks of chat group use and used a repeated-measures analysis of variance (ANOVA) with Huynh-Feldt correction for autocorrelation of adjacent time points to examine whether there was significant change over time in the number of days of practice completed and online chat group use.

We used an intent-to-treat analysis that included all participants regardless of whether they completed or dropped out of the intervention. We used the more conservative method of last observation carried forward to insert missing data immediately postintervention and during the 4 weeks of follow-up. We implemented repeated-measures ANOVA and reported effect size as partial eta-squared (*η*^2^_p_) using SPSS, version 25 (IBM Corp, Armonk, NY). We also computed a depression-change score from the baseline to the 4-week follow-up to be used as the dependent variable in subsequent linear regressions.

Using linear regression analysis controlling for baseline levels of depression, we examined whether change in depression symptoms from baseline to 4 weeks postintervention was predicted by the number of days of practice during the intervention or the number of weeks of group chat use.

## Results

### Participant Characteristics

As shown in Table 1, participants were primarily female, young adults, and college educated and all Caucasian. Thus, the samples in studies 1 and 2 were similar and uniform.

**Table 1:** Characteristics of participants.

Characteristic	Study 1 (n=22)	Study 2 (n=95)
Women, n (%)	22 (100)	76 (80.0)
Age (years), mean (SD)	23.2 (1.1)	32.0 (9.85)
Education (years), mean (SD)	17.4 (3.3)	16.3 (2.1)
Ethnicity (Caucasian), n (%)	22 (100)	95 (100)

### Study 1

#### Dropout, Daily Practice, and Weekly Group Chat

**Figure 1:**
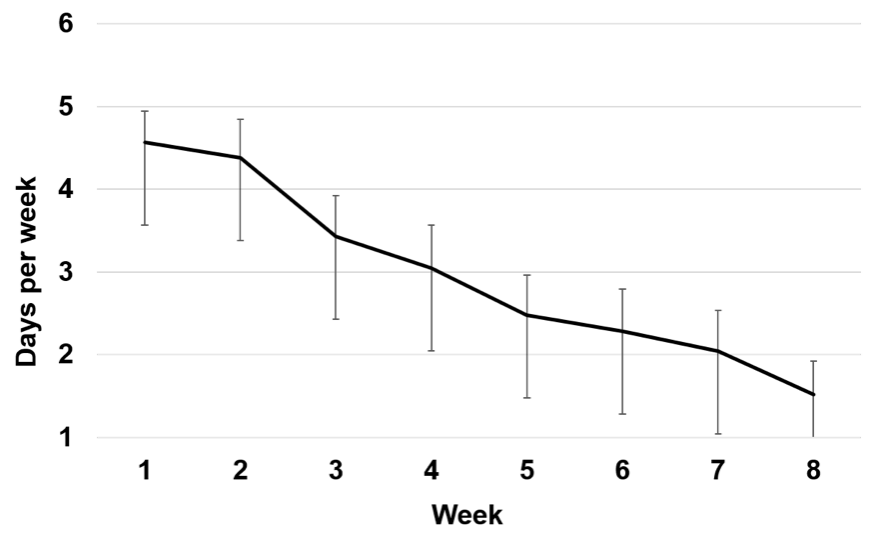
Number of days per week of mobile phone–delivered practices completed during the 8-week Ascend intervention during study 1. Error bars indicate standard error of the mean.

Dropout from the 8-week Ascend intervention was observed in 6 of the 22 participants (27%). For mobile phone–delivered exercises during the Ascend intervention, participants completed daily practices on 42% of the 56 days (mean 23.8 days; SD 14.2; range 1-49 days). A repeated-measures ANOVA, with Huynh-Feldt correction for autocorrelation of adjacent time points, showed that the mean number of days per week of practice decreased significantly (*F*_4.02_=15.02, *P<*.001, *η*^2^_p_=.43) from week 1 (mean 4.57 days [SD 1.72]) to week 8 (mean 1.52 [SD 1.83]) of the intervention (Figure 1). For the once-weekly group chat, participants participated for an average of 4.87 weeks (SD 3.12; range 0-8 weeks). Repeated-measures ANOVA, with Huynh-Feldt correction for autocorrelation of adjacent time points, showed that the percentage of participants who used the online chat group per week decreased significantly from week 1 (78%) to week 8 (52%; *F*_5.21_=2.65, *P*=.03, *η*^2^_p_=.11, *g*=.68). Participants reported a high and consistent level of the participant-therapist interaction across four time points during the 8-week intervention (mean 4.12).

#### Depression Symptoms

**Figure 2:**
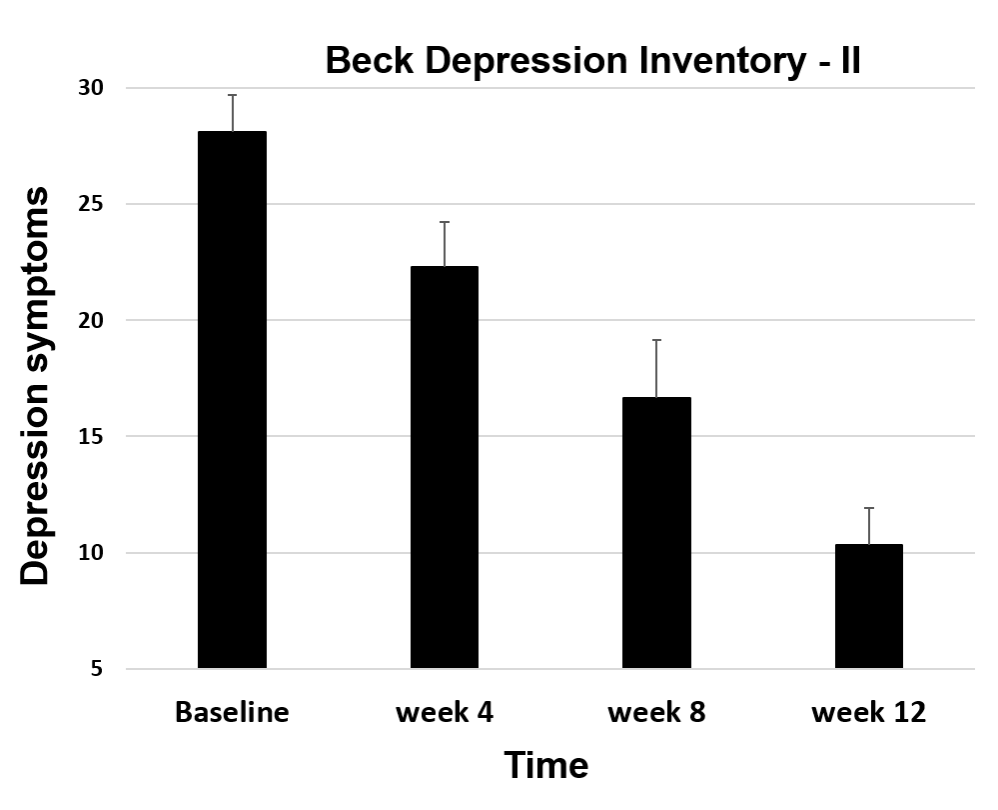
Depression symptoms during study 1 of the Ascend intervention. Error bars indicate standard error of the mean.

We used an intent-to-treat analysis that included all participants. Repeated-measures ANOVA for depression symptoms measured with the BDI-II revealed a significant reduction from before to after the intervention (mean delta=–8.55, SD 9.17; *F*_1,21_=19.11; *P*<.001; *η*^2^_p_=.48) as well as from before to 4 weeks after the intervention (ie, week 12 assessment; mean delta=–10.91, SD 10.35; *F*_1,21_= 24.44; *P*<.001; *η*^2^_p_=.54; Figure 2).

#### Predictors of Change in Depression Symptoms

**Figure 3:**
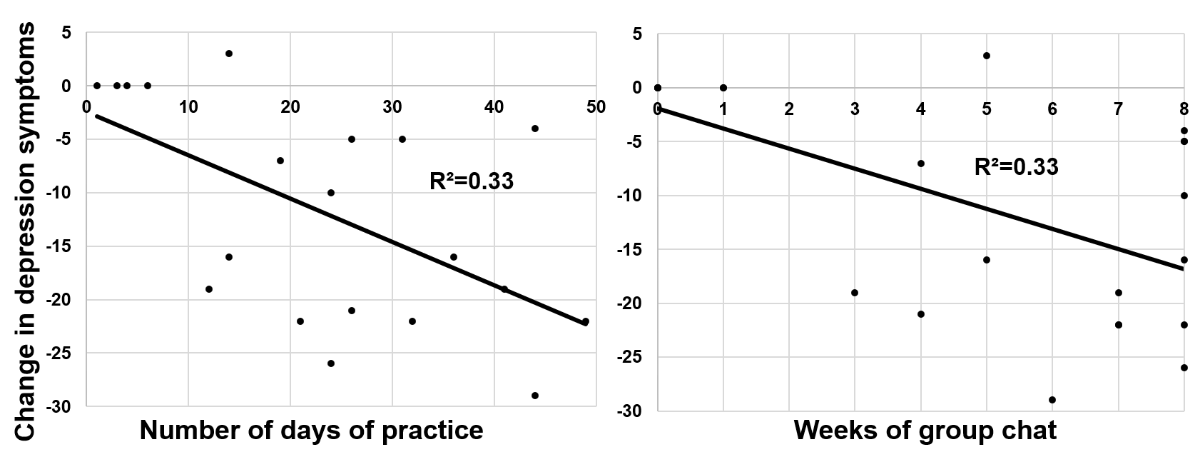
Daily practice and weekly group chat use during Ascend intervention predict pre-to-post change in depression symptoms in Ascend in study 1.

Using a linear regression controlling for baseline depression symptoms, we found that greater number of days of practice significantly predicted a reduction in depressive symptoms from the baseline to 4 weeks postintervention (BDI-II; ∆ *R*^*2*^=.38; ∆ *F*_1,20_=11.14; *P*=.004; unstandardized coefficient beta=–0.45, standard error of beta=0.14, 95% CI=–0.74 to –0.17; Figure 3). Similarly, we found that higher number of weeks of group chat use predicted a reduction in depressive symptoms from baseline to 4 weeks after the intervention (BDI-II; ∆ *R*^*2*^=.38; ∆ *F*_1,20_=12.30; *P*=.002; unstandardized coefficient beta=–2.06, SE of beta=.59, 95% CI=–3.29 to –0.83).

### Study 2

#### Dropout, Daily Practice, and Weekly Group Chat

**Figure 4:**
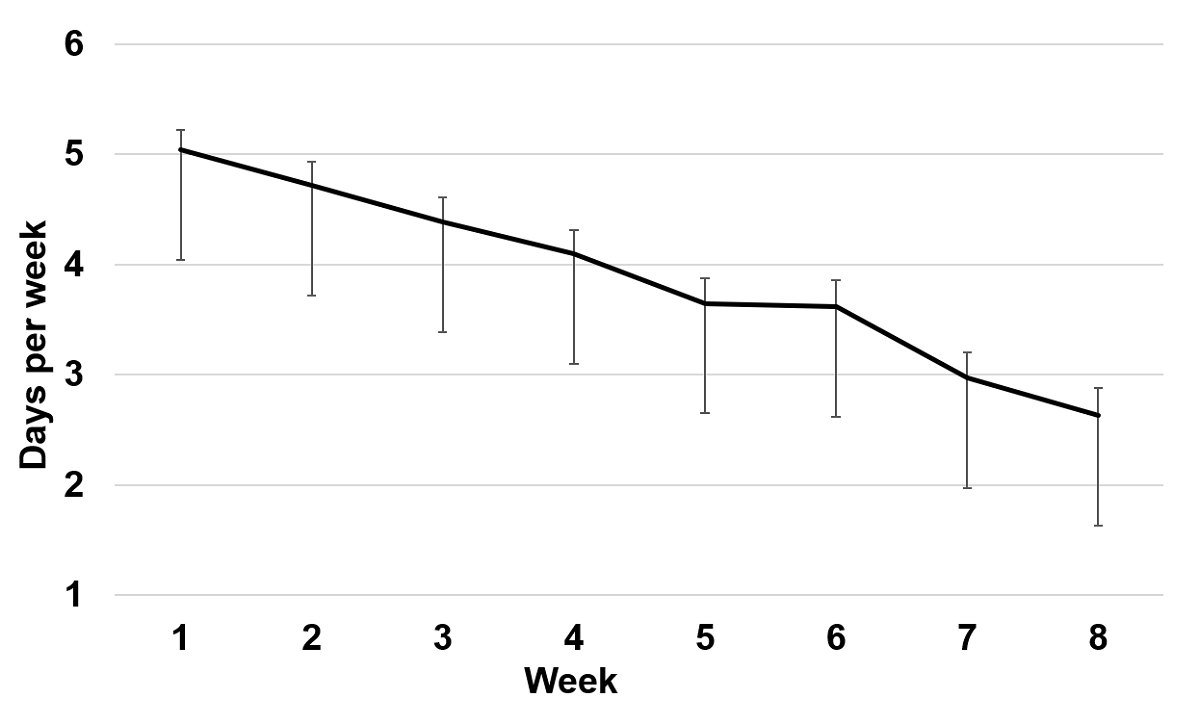
Number of days per week of mobile phone–delivered practices completed during the 8-week Ascend intervention during study 2. Error bars indicate standard error of the mean.

Dropout was noted in 14 of the 95 participants (15%). Participants completed the mobile phone–delivered exercises during the Ascend intervention on 55% of the days (mean 30.9 days; SD 14.1; range 1-56 days). A repeated-measures ANOVA, with Huynh-Feldt correction for autocorrelation of adjacent time points, showed that the mean number of days per week of practice decreased significantly (*F*_6.02, 535.33_=38.4; *P*<.001; *η*^2^_p_=.30) in a linear trajectory from week 1 (mean 5.04 days [SD 1.71]) to week 8 (mean 2.63 [SD 2.31]) of the intervention (Figure 4). For the once-weekly group chat, participants participated in an average of 5.81 weeks (SD 2.63; range 0-8 weeks). A repeated-measures ANOVA, with Huynh-Feldt correction for autocorrelation of adjacent time points, showed that the percentage of participants who used the online chat group per week decreased significantly from week 1 (85%) to week 8 (59%; *F*_5.59, 514.50_=6.09; *P*<.001; *η*^2^_p_=.06). Participants reported high and consistent levels of participant-therapist interaction across four time points during the 8-week intervention (mean=4.13).

#### Depression Symptoms

**Figure 5:**
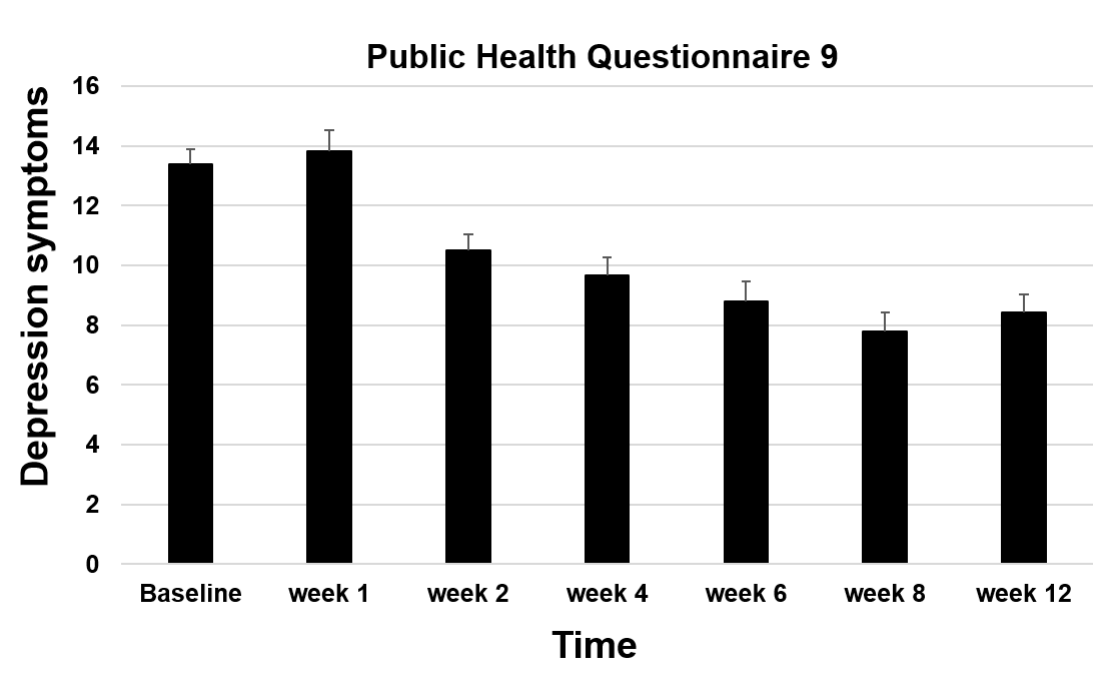
Depression symptoms during study 2 of the Ascend intervention. Error bars indicate standard error of the mean.

A repeated-measures ANOVA of depression symptoms measured with the PHQ-9 revealed a significant reduction from before to after the intervention (mean delta=–4.39, SD 5.07; *F*_1,94_=71.20; *P*<.001; *η*^2^_p_=.43) as well as from before to 4 weeks after the intervention (ie, week 12; *F*_1,94_=71.88; *P*<.001; *η*^2^_p_=.43; Figure 5).

#### Predictors Of Change in Depression Symptoms

**Figure 6:**
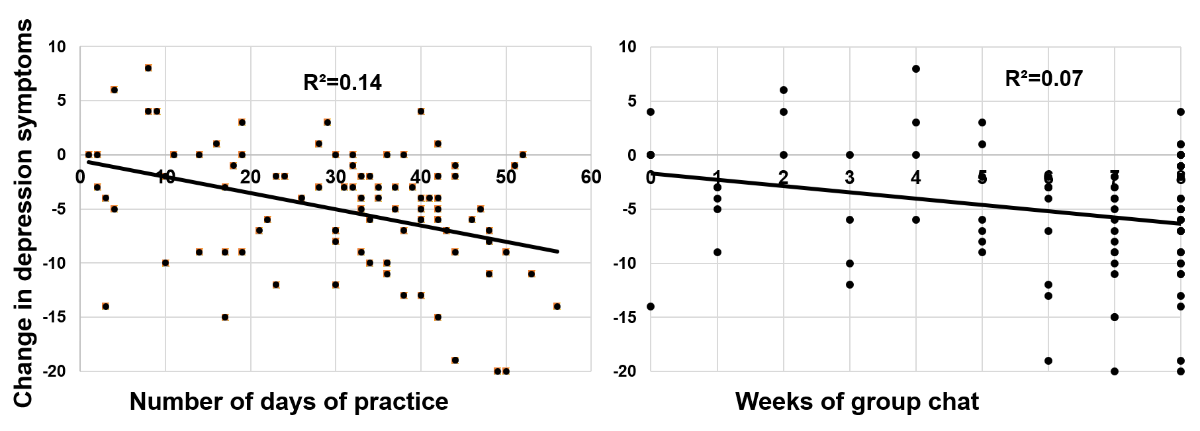
Daily practice and weekly group chat use during Ascend intervention predict pre-to-post change in depression symptoms in Ascend in study 2.

Using linear regression, we found that higher number of days of practice significantly predicted lower residual depression symptoms at 4 weeks postintervention (*∆R*^*2*^=.16, *F*_1,89_=16.46, *P*<.001; unstandardized beta=–0.028, standard error of beta=0.01, 95% CI=–0.041 to –0.014; Figure 6).

We also found that higher number of weeks of group chat use predicted reduced depressive symptoms at 4 weeks postintervention (∆ *R*^*2*^=.08, ∆ *F*_1,91_=9.92, *P*=.002; unstandardized coefficient beta=–0.63, standard error of beta=0.20, 95% CI=–1.03 to –0.23; Figure 6).

## Discussion

### Principal Findings

Findings from this study suggest that the mobile phone–delivered, therapist-supported Ascend intervention was associated with acceptable levels of dropout, linear decreasing daily practice, and weekly group chat use as well as significant decreases in depression symptoms in a sample of self-referred depressed individuals. Furthermore, the amount of daily practice and weekly group chat use during the intervention were associated with a reduction in depression symptoms from baseline to 4 weeks postintervention.

### Comparison with Prior Work

A recent RCT of CBT for major depression reported 33% dropout from mobile-delivered CBT (mobile phone or iPad) versus 30% dropout from computer-delivered CBT [25]. For comparison with nonmobile phone internet-delivered intervention for depression, a recent study [21] that employed a coach to guide people through online therapy for depressed patients reported a 37% dropout rate. Thus, the dropout rate observed in our pilot study (17%, 20/117 participants) was comparable to both other mobile phone– and internet-delivered interventions.

Daily practice of mobile phone–delivered exercises decreased linearly over the course of the 8-week Ascend intervention in both pilot studies. Given that the amount of daily practice significantly predicted a reduction in depression symptoms in each of the two pilot studies, this finding is disconcerting and strongly suggests that further effort is needed to investigate methods to support sustained practice, as it may lead to greater clinical improvement. This may involve introducing more novel types of practices or different modes of delivery of practices to maintain the participant’s attention and sustained engagement. Ongoing measurement of engagement during the intervention and tailoring the delivery to individual participants may help increase sustained practice. This could involve the use of individual participant feedback after each practice to direct what type of practice and modality is to be used when delivering subsequent practices. A brief motivational interview by the therapist may also identify and overcome obstacles to engagement. This is a component of internet interventions that would benefit from further research. Sustained engagement may likely increase the effect of the Ascend intervention and possibly reduce the probability of depression relapses.

Group chat, often referred to as synchronous text-based dialogue, is an increasingly important component of online mental health interventions. In our studies, the number of weeks of chat group use decreased significantly over the 8-week intervention. Like daily practice, group chat use was a significant predictor of a reduction in depressive symptoms. A recent review [26] found that interventions using one-on-one synchronous chat reduced clinical symptoms more than waitlists, but not more than treatment as usual (face-to-face and telephone counseling). Research has found that text-based chat communication involves approximately 50% fewer words than oral conversation [27]. In our study, however, the chat group, which included intervention participants and curation by the therapist, was only one component of the intervention. More research is needed to better understand the factors that encourage and inhibit group chat use in mobile phone–delivered interventions for depression.

The Ascend intervention was associated with a reduction in depression symptoms across two pilot studies. However, because we did not incorporate a comparison control group, we cannot rule out regression to the mean as a reason for the reduced depression symptoms. Nonetheless, our intent-to-treat analysis revealed a moderate effect (*g*=.43 to .54) for reduction of depression symptoms across the two pilot studies. Our findings are in line with the results from a recent meta-analysis [15] of mobile phone–delivered interventions for depression that found moderate effect sizes for intent-to-treat or complete outcome data trials (*g*=.40), CBT-based interventions (*g*=.53), and interventions that included mindfulness practices (*g*=.49). Importantly, subanalyses in this meta-analysis that focused on diagnosed mood disordered samples showed that mobile phone–delivered interventions were most effective for patients with self-reported mild-to-moderate levels of depression symptoms (*g*=.52). This is important because individuals with mild-to-moderate depression symptoms are most likely to have the capacity to use mobile phone and other online interventions.

Identification of predictors of better outcomes is essential for determining how to refine and optimize a clinical intervention. We found that higher number of days of completing mobile phone–delivered practices predicted a greater reduction in self-reported depression symptoms. Variance in the reduction of depression symptoms explained by practice during the 8-week Ascend intervention ranged from 7% to 38%. Additionally, in both pilot studies, we found that greater chat group use predicted reduced depression symptoms. Although these findings are promising, they also suggest that there likely are other factors that predict outcome and may interact with subgroups of patients. It will be important to determine which specific practices and what amount of group chat use are most effective for reducing depression symptoms.

Identifying patient characteristics that predict the impact of online interventions on depression symptoms is essential. A well-controlled study comparing self-guided internet-delivered interpersonal psychotherapy, CBT, and MoodGYM found that female gender, lower mastery, and lower dysfunctional attitudes predicted lesser depression symptoms following 4 weeks of treatment and 6 months of follow-up regardless of the intervention type [28]. Understanding how these patient characteristics interact with daily practice and group chat use should be examined in future studies.

Given that novelty captures attention, one approach is to explore on an individual participant level whether introducing new modalities of delivery of didactic content and practices might recapture attention and dedication to practice. An algorithm could be used to detect when the number of days of practice begins to decrease and then introduce new videos, practices, or communication from other participants and psychotherapist to buttress against further reduction in practice. Another method would be to show each individual their own week-to-week relationship between practice and depression symptoms to further motivate engagement with the Ascend program.

### Limitations

Participants in this study were self-referred, and thus, there may be a sampling bias in that these individuals were more motivated to participate than the normative population of depressed individuals. Furthermore, the sample consisted of 80%-100% women, which is also not representative of the population of individuals with depression in clinical settings or the community. This pilot study did not include any control group with or without an active comparison intervention, which limits the inferences that can be made regarding the efficacy or comparison of the effect sizes between the Ascend program and other interventions. Future studies will benefit from comparison of Ascend to gold-standard psychosocial (eg, CBT) and pharmacological interventions as well as a control group without an active intervention. Although this study provides initial evidence of a reduction in depression symptoms, future studies should include diagnostic assessment of participants. In addition to changes in clinical symptoms, it will be important to measure putative mediators of the Ascend intervention related to mindfulness meditation (eg, mindfulness skills) and CBT (eg, cognitive reappraisal). Future studies might consider determining whether the amount (eg, duration in minutes and frequency of events) or type of therapist-patient interaction (eg, chat, phone, and email) predicts intervention outcome.

### Conclusions

The overall finding from this pilot study is that the 8-week online Ascend intervention was associated with reduced depression symptoms. However, more rigorous controlled trials that compare the Ascend intervention with both other online mental health interventions and gold-standard clinical interventions for major depression are needed.

## Abbreviations

ANOVA: analysis of variance
BDI-II: Beck Depressive Inventory-II
CBT: cognitive behavioral therapy
HIPAA: Health Insurance Portability and Accountability Act
PHQ-9: Patient Health Questionnaire 9-item version
RCT: randomized controlled trial
WHO: World Health Organization
